# Cinnamoyl-Oxaborole Amides: Synthesis and Their in Vitro Biological Activity

**DOI:** 10.3390/molecules23082038

**Published:** 2018-08-15

**Authors:** Maureen Gumbo, Richard M. Beteck, Tawanda Mandizvo, Ronnett Seldon, Digby F. Warner, Heinrich C. Hoppe, Michelle Isaacs, Dustin Laming, Christina C. Tam, Luisa W. Cheng, Nicole Liu, Kirkwood M. Land, Setshaba D. Khanye

**Affiliations:** 1 Faculty of Science, Department of Chemistry, Rhodes University, Grahamstown 6140, South Africa; mgumbo1@gmail.com (M.G.); richmbi1@yahoo.com (R.M.B.); tmandizvo@gmail.com (T.M.); 2 Drug Discovery and Development Centre (H3-D), Department of Chemistry, University of Cape Town, Rondebosch 7701, South Africa; ronnett.seldon@uct.ac.za; 3 MRC/NHLS/UCT Molecular Mycobacteriology Research Unit, Department of Pathology, University of Cape Town, Rondebosch 7701, South Africa; Digby.Warner@uct.ac.za; 4 Institute of Infectious Disease and Molecular Medicine, University of Cape Town, Rondebosch 7701, South Africa; 5 Faculty of Science, Department of Biochemistry and Microbiology, Rhodes University, Grahamstown 6140, South Africa; h.hoppe@ru.ac.za; 6 Centre for Chemico- and Biomedicinal Research, Rhodes University, Grahamstown 6140, South Africa; m.isaacs@ru.ac.za (M.I.); dustinlaming89@gmail.com (D.L.); 7 Foodborne Toxin Detection and Prevention Research Unit, Agricultural Research Service, United States Department of Agriculture, Albany, CA 94710, USA; christina.tam@ars.usda.gov (C.C.T.); luisa.cheng@ars.usda.gov (L.W.C.); 8 Department of Biological Sciences, University of the Pacific, Stockton, CA 95211, USA; n_liu@u.pacific.edu (N.L.); kland@PACIFIC.EDU (K.M.L.)

**Keywords:** benzoxaboroles, cinnamic acids, trichomoniasis, trypanosomiasis, *Mycobacterium tuberculosis*

## Abstract

Due to the increased interest in their application in the treatment of infectious diseases, boron-containing compounds have received a significant coverage in the literature. Herein, a small set of novel cinnamoly-oxaborole amides were synthesized and screened against nagana *Trypanosoma brucei brucei* for antitrypanosomal activity. Compound **5g** emerged as a new hit with an in vitro IC_50_ value of 0.086 μM against *T. b. brucei* without obvious inhibitory activity against HeLa cell lines. The same series was also screened against other human pathogens, including *Mycobacterium tuberculosis*, the causative agent of tuberculosis (TB), for which moderate to weak activity (10 to >125 μM) was observed. Similarly, these compounds exhibited moderate activity against the human protozoal pathogen *Trichomonas vaginalis* with no observed effect on common microbiome bacterial species. The cross-species inhibitory activity presents the possibility of these compounds serving as broad-spectrum antibiotics for these prevalent three human pathogens.

## 1. Introduction

Globally, the major tropical infections malaria, tuberculosis (TB), trypanosomiasis, and leishmaniasis remain a serious public health concern. Collectively, these infectious diseases are responsible for more than 2.2 million deaths in developing countries annually [1]. Despite it being a preventable infectious disease, in 2015, approximately 1.8 million people died from TB infection [2]. A complete eradication of TB remains an incomplete task since drugs to treat these diseases are facing several limitations including reduced efficacy, poor safety, and affordability [3]. A further complication is the emergence of multidrug resistant *Mycobacterium tuberculosis* against clinically proven drugs, which has made the development of new therapies a demanding task for effective management and control of TB. Additionally, TB is considered a primary killer in HIV-positive individuals, and the World Health Organization (WHO) reported that people living with HIV are at higher risk of developing TB compared to healthy individuals [2]. In recent years, there have been reports of an overlap of endemic regions between TB and parasitic diseases, which may result in co-infections of these diseases in the affected population [4].

**Figure 1:**
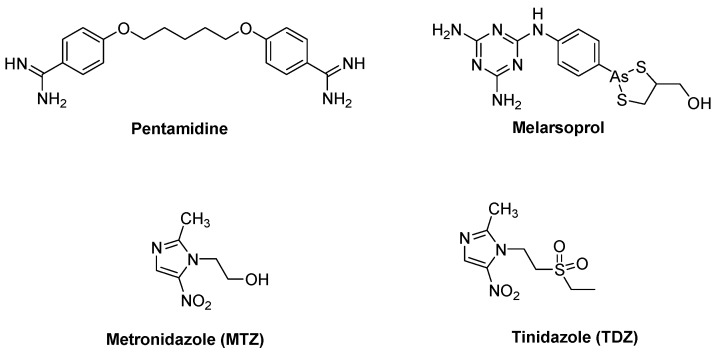
Chemical structures of drugs for treatment of sleeping sickness and trichomoniasis.

Human African trypanosomiasis (HAT), commonly referred to as a sleeping sickness [5], is a fatal and debilitating disease which is included in the list of 17 neglected tropical diseases (NTDs) prioritized by WHO [6,7,8]. Approximately 70 million people mainly residing in the remote areas of sub-Saharan Africa are at risk of contracting the disease [9,10]. Human African trypanosomiasis is caused by *Trypanosoma brucei* sub-species, namely *T. b. rhodesiense* and *T. b. gambiense* [11,12]. It is spread from one person to another via the bite of an infected tsetse fly [13]. Although the causal agents of HAT have been known for over a century, only four drugs, pentamidine, melarsoprol (Figure 1), suramin, and eflornithine, are registered for the treatment of this disease [14]. These long-discovered drugs have very poor oral bioavailability; as such, they are only administered intravenously, which is expensive [15] and requires expertise and facilities that are inaccessible in disease stricken rural areas [16], hampering treatment implementation [17]. In addition, these drugs also have serious side effects [18] including nephrotoxicity, hypertension, anemia, and neuropathy [19]. Moreover, there are growing concerns over the development of the drug-resistant infections against these drugs [20].

Similarly, individuals affected by parasitic diseases and bacterial infections are also exposed to sexually transmitted diseases [3]. Each year >340 million new cases of sexually transmitted bacterial and protozoal infections (STIs) occur globally [21,22]. Included in the list of STIs is human trichomoniasis, a non-viral disease that is caused by *Trichomonas vaginalis*. Contrary to infections such as chlamydia, gonorrhea, and syphilis, trichomoniasis receives less attention in terms of control efforts despite it being the most common disease of all [23]. Problems associated with trichomoniasis include premature rupture of membranes during pregnancy, premature birth, low birth weight, and enhancing the acquisition of HIV [24], an infectious virus posing a major threat especially in Africa. Nitroimidazoles, metronidazole, and tinidazole (Figure 1), are preferred drugs for treatment of *T. vaginalis* infections [25]. With the emerging resistance of *T. vaginalis* against metronidazole, there is no well-established alternative therapy available.

**Figure 2:**
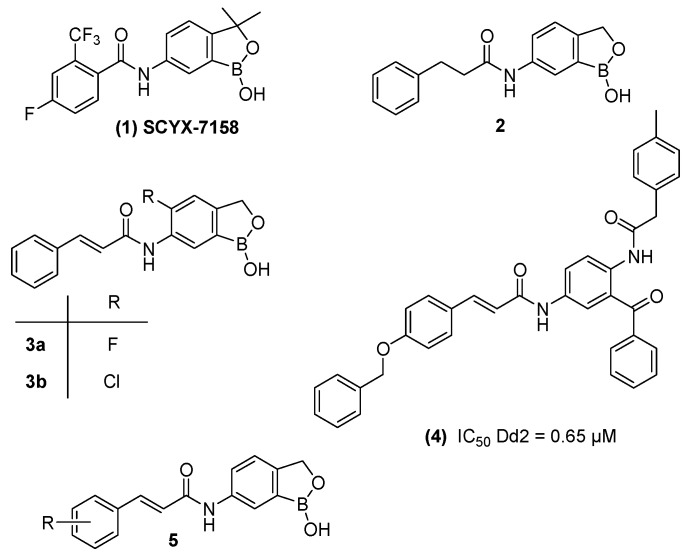
Chemical structures of antifungal, antiplasmodial, and trypanocidal benzoxaboroles including the proposed cinnamoyl-oxaborole amides (**5**).

Considering their attractive therapeutic and biological profile, in recent years, boron-containing compounds have received significant attention and are featuring prominently in literature [5,26,27,28,29,30,31,32,33]. Typical examples of these compounds are benzoxaboroles, which are cyclic boronic acids with robust stability under elevated acidic and basic conditions. Initially discovered by Torssell in 1957 [34], benzoxaboroles have been hailed as potent anti-infective agents [26,27,28,29] that are non-toxic and possess desirable chemical properties as well as pharmacokinetic properties [31]. Recent endeavors aimed at discovering novel trypanocidal agents have culminated in new chemical entities (NCEs) entering clinical trials [35], one of which is SCYX-7158 (**1**)—a benzoxaborole-based compound [36] (Figure 2). Due to high attrition rates during clinical development of NCEs [37], it is imperative to replete the trypanocidal drug development pipeline with numerous and structurally diverse benzoxaborole-based leads. Typical examples of other promising novel benzoxaborole-containing compounds include compound **2** (Figure 2), which showed excellent trypanocidal activity with in vitro IC_50_ value of 0.14 μg/mL against *T. b. brucei* S427 [38]. Furthermore, compounds **3a** and **3b** (Figure 2) are two examples of cinnamic acid-based benzoxaborole derivatives, which have been studied as antifungal agents [39].

More importantly, cinnamic acids are a class of compounds, which have received considerable attention as promising anti-infective agents [40]. Several studies have shown that cinnamic acid and their corresponding phenolic derivatives exhibit a broad spectrum of biological activities including antiproliferative, antioxidant, antiviral, antimicrobial, antimalarial, and anti-cardiovascular activities [40,41,42]. For example, Wiesner and co-workers [43] reported the antimalarial activity of 2,5-bisacylaminobenzonephone (Figure 2, Compound **4**) obtained by replacing a 3-phenylpropanamide unit with 3-phenylprop-2-enamide unit. This prompted us to explore the biological activity of compounds containing the-prop-2-enamide unit coupled with 6-aminobenzoxaborole, which is an appealing scaffold with antiprotozoal properties. Except the cinnamic acid-based benzoxaborole derivatives (Figure 2, Compounds **3a** and **3b**), there is no previous literature report on this class of compounds as potential antiprotozoal agents. Hence, herein we wish to report on the synthesis and in vitro biological evaluation of novel cinnamoyl-oxaborole amides (Figure 2, Compound **5**).

## 2. Results and Discussion

### 2.1. Chemistry

**Scheme 1:**
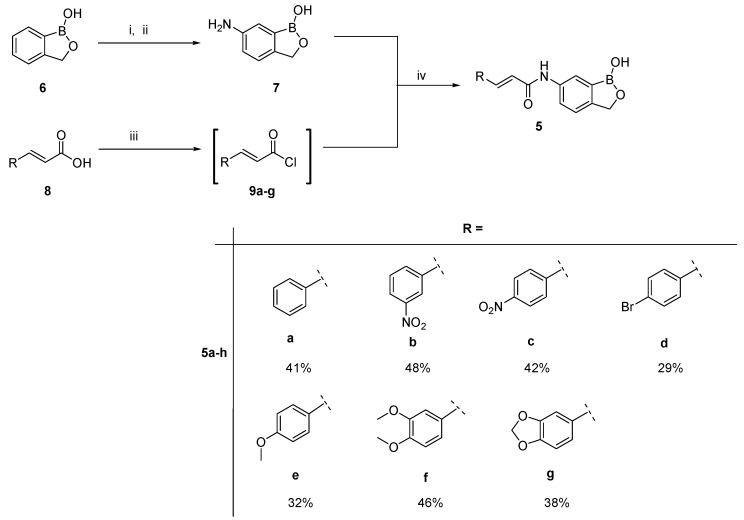
Synthesis of cinnamoyl˗benzoxaborole amides **5a**–**g**. Reagents and conditions: (i) 99% HNO_3_, −30 °C, 3 h; (ii) H_2_, Pd/C, r.t, 2 h; (iii) DCM, Et_3_N, POCl_3_ and then (iv) DMAP.

The synthesis of all intermediates and target compounds is presented in Scheme 1 below. Briefly, the commercially accessible benzoxaborole 6 was treated with nitric acid at −30 to −40 °C to form 6-nitrobenzoxaborole intermediate, and subsequently reduced to form 6-aminobenzoxaborole **7** under hydrogen atmosphere using palladium on carbon catalyst [44]. With the 6-aminobenzoxaborole **7** in hand, we turned our attention to the activation of cinnamic acids **8** for coupling with **7** to form the desired target cinnamoly-oxaborole amides **5a**–**g**. Initially, we tried in situ activation of the carboxylic acid group of cinnamic acids for amidation using different coupling agents such as CDI, EDCI, HOBt, HOSu [45], and upon addition of 6-aminobenzoxaborole **7** these coupling agents all gave traces of desired products. We then opted for the conversion of cinnamic acids to their more reactive cinnamoyl chloride derivatives [46]. The common method of converting carboxylic acids to acyl chlorides, which makes use of SOCl_2_ [47] was attempted, and upon coupling with 6-aminobenzoxaborole **7**, the reaction gave poor yields, and in some cases the desired products could not be isolated.

We then adopted an alternative method using POCl_3_ in the presence of Et_3_N, an organic base. The presence of an organic base to maintain a basic environment has been reported to be important for acylation reactions of this type [48]. Thus, cinnamic acids **8** were treated with phosphoryl chloride under nitrogen atmosphere to form in situ the corresponding cinnamoyl chloride intermediates **9a**–**g**. 6-Aminobenzoxaborole **7** was then acylated with the intermediates **9a**–**g** to form targeted compounds **5a**–**g** in 29 to 46% yields.

The synthesized cinnamoyl-oxaboroles (Scheme 1, **5a**–**g**) were characterized using ^1^H and ^13^C nuclear magnetic resonance (NMR) as well as high resolution mass spectrometry (HRMS). The ^1^H-NMR spectra of all compounds showed a signal at δ *ca* 10.27 ppm assigned to B-OH. The peak at δ *ca* 9.24 ppm is assigned to -NH of the amide bond. Two doublets (*J* = 15.0 and 15.6 Hz) at δ *ca* 6.87 and 7.60 ppm are attributed to the two -CH of prop-2-enamide. These coupling constants are consistent with a trans geometry at the double bond. The signal at δ *ca* 4.96 ppm is assigned to the oxaborole -CH_2_. The ^13^C-NMR spectra of all compounds confirmed the skeletal structures consistent with proposed compounds **5a**–**g**. The non-appearance of the carbon adjacent to the boron atom was noted, which is a common trend in benzoxaborole-containing compounds [49]. Analysis of the HRMS revealed molecular ion peaks agreeing with overall molecular structures of achieved compounds. The purity of all compounds was determined to be greater than 96% using qualitative high performance liquid chromatography (HPLC) (reversed phase).

### 2.2. Pharmacology

**Figure 3:**
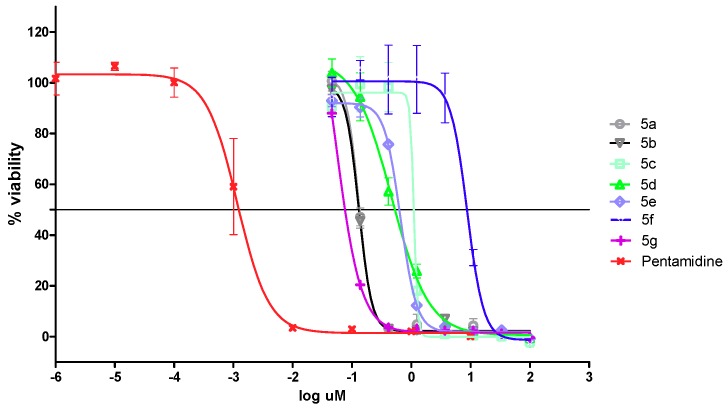
Percentage viability of the *T. b. brucei* parasites determined at different compound concentrations and corresponding IC_50_ values of compounds **5a**–**g**.

All target compounds were screened for in vitro inhibitory activity against the 427 strain of *T. b. brucei*, the *T. vaginalis* parasite, and *M. tuberculosis* H37Rv. The cytotoxicity of all compounds was assessed against human cervix adenocarcinoma (HeLa) cell line and emetine was used as a control. At a 20 μM concentration of each compound, none of the compounds showed any toxic effects to the HeLa cell line, and >80% cell viability was maintained during a 48 h exposure to the compounds (Table 1. At this concentration, all tested compounds exhibited greater than 90% growth inhibition of trypanosome parasites. Based on the single concentration screening data, the IC_50_ values were determined for compound **5a**–**g**; the results are summarized in Figure 3 and Table 2. The cinnamoly-oxaborole amides showed promising antitrypanosomal activity with IC_50_ values in the low micromolar to sub-micromolar range. Despite being slightly less active compared to compounds **1** and **2**, compounds **5a**–**e** showed good activity with IC_50_ values below 1 μM and compound **5g** emerged as the most active compound with IC_50_ value of 0.086 μM against the *T. b. brucei* parasite. Pentamidine was used as a positive control antitrypanosomal drug (IC_50_ = 1.2 nM).

Consistent with other studies [43,50,51,52,53,54] our data seem to suggest that linking of the cinnamoyl framework with bioactive scaffolds lead to compounds showing superior activity. The more active compounds also displayed excellent selectivity for the trypanosomal parasites over the human HeLa cell line. Thus far, the preliminary structure-activity relationship (SAR) suggested that mono-substituted aryl moieties are preferred for activity. More importantly, bicyclic aryl frameworks as observed for compound **5g** emerged as promising ring systems, which are worth further exploration.

Interestingly, inhibitory activity profiles for both mycobacteria and trichomonad parasites revealed similar SAR, suggesting that the cellular targets of these compounds may be similar, even though mycobacteria are prokaryotic and trichomonads are eukaryotic pathogens. Also, given that trichomonad parasites co-exist with a mucosal microbiome, screening of these compounds showed no detectable effects on normal flora bacterial growth in vitro. Taken together, these compounds may present a new scaffold for drug discovery against human trypanosomiasis, tuberculosis, and human trichomoniasis.

Target compounds **5a**–**g** were also evaluated for antimycobacterial activity in vitro. To this end, the compounds were screened in a broth microdilution assay against *Mycobacterium tuberculosis* H37Rv (*Mtb*) using rifampicin as a standard. The antimycobacterial activities are reported as minimum inhibitory concentration (MIC_99_) required to inhibit mycobacterial growth by 99%. Antimycobacterial activities are incorporated in Table 1. More than 50% of the compounds exhibited antimycobacterial activities, although these were moderate with respect to activity of the standard, rifampicin (MIC_99_ 0.003 μM). Starting from compound **5a** (MIC_99_ 61.8 μM), di-substitution of the aryl moiety resulted in the loss of activity (e.g., **5f**, MIC_99_ ˃ 50 μM), while mono-substitution enhances activity (e.g., **5e**, MIC_99_ 18.7 μM). The para-substituted aryl analogues showed the best activities (**5d**, MIC_99_ 10.7 μM).

## 3. Materials and Methods

### 3.1. General Information

All chemicals and solvents used were purchased from Sigma-Aldrich (Pty) Ltd (Johannesburg, South Africa) and Merck (Pty) Ltd (Johannesburg, South Africa) and were used without further purification. Reactions were monitored by analytical thin layer chromatography (TLC) using Merck F254 silica gel plates (Merck, Johannesburg, South Africa) supported on aluminum sheets and the plates were visualized under ultraviolet (UV 254 and 366 nm) light and in iodine flasks. Where necessary, the crude compounds were purified by a silica gel column chromatography using Merck Kieselgel 60 Å:70–230 (0.068–0.2 mm) silica gel mesh (Merck, Johannesburg, South Africa). The ^1^H and ^13^C-NMR spectra were recorded on Bruker Biospin 300, 400 or 600 MHz spectrometers, and were referenced internally using residual solvent signals of deuterated DMSO-*d_6_*: 2.50 ppm for ^1^H and 39.5 ppm for ^13^C-NMR, or deuterated chloroform CDCl_3_: 7.26 ppm for ^1^H and 77.2 ppm for ^13^C-NMR at ambient temperature. The high-resolution mass spectrometric data (HRS-MS) of the final compounds was recorded on Waters Synapt G2 Mass Spectrometer (Stellenbosch University, Stellenbosch, South Africa) using electron impact (EI) ionization in the positive ionization mode. The starting 6-aminobenzoxaborole **7** was synthesized from the commercial 6-nirobenzoxaborole **6** (see Supplementary Materials) as previously described in the literature [31,55]. Purity was determined by HPLC (Agilent, Santa Clara, CA, USA), and all compounds were confirmed to have a purity of >95%. The chromatographic system consisted of an Agilent HP1100 LC-MSD (Agilent, Santa Clara, CA, USA) and equipped with a quaternary pump, in-line degasser, DAD detector, 1100 MSD and ChemStation for collection and analysis of data. A ZORBAX Elipse Plus C18 4.6 i.d. × 150 mm × 5 μm column was used for reversed-phase HPLC analysis. A mobile phase consisting of a mixture of aqueous solution of monobasic sodium phosphate 0.01 M and acetonitrile (90:10) on isocratic elution mode was used. Five different concentrations (5–500 μg/mL) of samples to be analyzed were made, filtered using 0.45 μm Millipore filters before their injection.

### 3.2. General Synthetic Procedure for the Cinnamic Acid-Benzoxaborole Hybrids, ***5a**–**g***

A mixture of cinnamic acids **2** (0.5 mmol) and Et_3_N (1.2 mmol) in anhydrous CH_2_Cl_2_ (1.5 mL) under N_2_ atmosphere was stirred at 0 °C for 5 min, then POCl_3_ (0.5 mmol) in anhydrous CH_2_Cl_2_ (1 mL) was added and the resulting mixture allowed to stir at room temperature for 30 min. When the acids were completely consumed (TLC), a solution of DMAP (0.15 mmol) and 6-aminobenzo[c] [1,2]oxaborol-1(3H)-ol (0.6 mmol) in anhydrous CH_2_Cl_2_ (0.5 mL) was added dropwise and the mixture stirred overnight at room temperature. Upon completion, the reaction mixture was washed sequentially with ice-cold water (10 mL), 10% aqueous HCl (10 mL), saturated aqueous NaHCO_3_ solution (10 mL), brine, and dried over anhydrous MgSO_4._ The solvent was removed in vacuo to give a solid residue, which was purified by flash column chromatography to afford the pure products, **5a**–**g**.

*(E)-N-(1-Hydroxy-1,3-dihydrobenzo[c][1,2]oxaborol-6-yl)cinnamamide* (**5a**). Off white solid. Yield = 41%. m.p.: 182–184 °C. ^1^H-NMR (300 MHz, DMSO-*d_6_*) δ_H_ (ppm): 10.28 (1H, s, B-OH), 9.25 (1H, s, NH), 8.11 (1H, d, *J* = 1.8 Hz, Ar-H9), 7.78 (1H, dd, *J* = 1.8 and 6.3 Hz, Ar-H10), 7.65–7.56 (3H, m, Ar-H7/H6/H2), 7.46–7.34 (4H, m, Ar-H11, Ar-H5, Ar-H4, Ar-H3), 6.90 (1H, d, *J* = 15.6 Hz, H8), 4.96 (2H, s, H12). ^13^C-NMR (75 MHz, DMSO-*d_6_*) δ (ppm): 163.6, 149.4, 140.4, 138.6, 135.3, 130.1, 129.6, 128.2, 122.9, 122.8, 122.1, 121.5, 70.2. *m/z* (ESI-MS) found 280.1146 [M + H]^+^, expected 279.1067. HPLC purity = 99.3%, retention time = 1.01 min.

*(E)-N-(1-Hydroxy-1,3-dihydrobenzo[c][1,2]oxaborol-6-yl)-3-(3-nitrophenyl)acrylamide (***5b**). Yellow solid. Yield = 48%. m.p.: 176–178 °C. ^1^H-NMR (300 MHz, DMSO-*d_6_*) δ_H_ (ppm): 10.23 (1H, s, B-OH), 8.09 (1H, d, *J* = 3.0 Hz, Ar-H9), 8.77 (1H, dd, *J* = 3.0 and 6.0 Hz, Ar-H10), 7.52 (1H, d, *J* = 15.0 Hz, H7), 7.35 (1H, d, *J* = 6.0 Hz, Ar-11), 7.20–7.13 (2H, m, H4, H2),7.03–6.96 (1H, m, Ar-H6), 6.90–6.82 (1H, m, Ar-H5), 6.73 (1H, d, *J* = 15.0 Hz, H8), 4.94 (2H, s, H12). ^13^C-NMR (75 MHz, DMSO-*d_6_*) δ_C_ (ppm):164.5, 149.4, 148.7, 140.4, 138.9, 129.9, 124.1, 122.9, 122.4, 121.7, 121.1, 109.4, 107.0, 102.2, 70.4. *m/z* (ESI-MS) found 324.1046 [M]^+^, expected 324.0918. HPLC purity = 92.5%, retention time = 1.43 min.

*(E)-N-(1-Hydroxy-1,3-dihydrobenzo[c][1,2]oxaborol-6-yl)-3-(4-nitrophenyl)acrylamide* (**5c**). Yellow solid. Yield = 42%. m.p.: 176–180 °C.^1^H-NMR (600 MHz, DMSO-*d_6_*) δ_H_ (ppm): 10.42 (1H, s, B-OH), 9.29 (1H, s, H10), 8.29 (2H, d, *J* = 9.0 Hz, Ar-Hf), 8.10 (1H, d, *J* = 1.5 Hz, Ar-H7), 7.90 (2H, d, *J* = 9.0 Hz, Ar-He), 7.77 (1H, dd, *J* = 1.8, 9.0 Hz, Ar-H5), 7.70 (1H, d, *J* = 15.6 Hz, Hb), 7.39 (1H, d, *J* = 8.4 Hz, Ar-H4), 7.05 (1H, d, *J* = 15.0 Hz, Hc), 4.96 (2H, s, H3). ^13^C-NMR (151 MHz, DMSO-*d_6_*) δ_C_ (ppm): 163.3, 149.6, 148.1, 141.8, 138.4, 138.0, 129.2, 127.2, 124.6, 122.8, 122.2, 121.6. 70.3. ESI-HRMS *m/z* calcd. for C_16_H_13_BN_2_O_5_ 324.0918, found 325.1005 [M + H]^+^. HPLC purity = 99.1%, retention time = 1.33 min.

*(E)-3-(4-Bromophenyl)-N-(1-hydroxy-1,3-dihydrobenzo[c][1,2]oxaborol-6-yl)acrylamide* (**5d**). Off white solid. Yield = 29%. m.p.: 206–210 °C. ^1^H-NMR (300 MHz, DMSO-*d_6_*) δ_H_ (ppm):10.33 (1H, s, B-OH), 9.28 (1H, s, H10), 8.32 (1H, d, *J* = 2.8 Hz, Ar-H7), 8.06 (1H, dd, *J* = 3.0, 6.0 Hz, Ar-H5), 7.61–7.51 (4H, m, Ar-He, -Hf), 7.53 (1H, d, *J* = 15.0 Hz, Hb), 7.36 (1H, d, *J* = 9.0 Hz, Ar-4), 6.87 (1H, d, *J* = 15.0 Hz, Hc), 4.94 (2H, s, H3). ^13^C-NMR (75 MHz, DMSO-*d_6_*) δ_C_ (ppm): 163.9, 149.6, 139.3, 139.3, 138.3, 134.4, 132.4, 130.2, 123.4, 123.0, 122.2, 121.6, 70.2. ESI-HRMS *m/z* calcd. for C_16_H_13_BBrNO_3_ 357.0172, found 358.0250 [M + H]^+^. HPLC purity = 98.4%, retention time = 0.67 min.

*(E)-N-(1-Hydroxy-1,3-dihydrobenzo[c][1,2]oxaborol-6-yl)-3-(4-methoxyphenyl)acrylamide* (**5e**). Off white solid. Yield = 32%. m.p.: 190–192 °C. ^1^H-NMR (300 MHz, DMSO-*d_6_*) δ_H_ (ppm): 10.19 (1H, s, B-OH), 9.25 (1H, s, NH), 8.09 (1H, d, *J* = 1.2 Hz, Ar-H9), 7.75 (1H, dd, *J* = 1.8 and 6.6 Hz, Ar-H10), 7.54 (1H, d, *J* = 15.0 Hz, H7), 7.36 (1H, d, *J* = 9.0 Hz, Ar-11), 7.01 (2H, d, *J* = 9.0 Hz, Ar-H2, H6), 6.97 (2H, d, *J* = 9.0 Hz, Ar-H5, H3), 6.74 (1H, d, *J* = 15.0, H8), 4.95 (2H, s, H12), 3.15 (3H, s, H4’). ^13^C-NMR (75 MHz, DMSO-*d_6_*) δ_C_ (ppm): 168.4, 164.4, 161.1, 149.3, 138.9, 130.5, 130.57, 130.0, 127.8, 127.7, 120.6, 115.1, 70.5, 56.1. *m/z* (ESI-MS) found 310.1252 [M + H]^+^, expected 309.1172. HPLC purity = 99.5%, retention time = 0.95 min.

*(E)-3-(3,4-Dimethoxyphenyl)-N-(1-hydroxy-1,3-dihydrobenzo[c][1,2]oxaborol-6-yl)acrylamide* (**5f**). Pale yellow solid. Yield = 46%. m.p.: 188–190 °C. ^1^H-NMR (300 MHz, DMSO-*d_6_*) δ_H_ (ppm): 10.20 (1H, s, B-OH), 9.35 (1H, s, NH), 8.05 (1H, d, *J* = 2.6 Hz, Ar-H9), 7.71 (1H, dd, *J* = 3.0 and 6.0 Hz, Ar-H10), 7.51 (2H, d, *J* = 15.6 Hz, H7), 7.35 (1H, d, *J* = 9.0 Hz, Ar-11), 7.20–7.13 (2H, m, H5, H2), 6.98 (1H, d, *J* = 9.0 Hz, H6), 6.73 (1H, d, *J* = 15.0, H8), 4.93 (2H, s, H12), 3.79 (6H, s, H4’, H3). ^13^C-NMR (75 MHz, DMSO-*d_6_*) δ_C_ (ppm): 168.9, 164.9, 151.2, 151.6, 149.7, 141.2, 138.8, 128.1, 127.7, 123.4, 122.7, 122.5, 122.0, 120.6, 110.9, 56.4, 56.3. *m/z* (ESI-MS) found 340.1357 [M + H]^+^, expected 339.1278. HPLC purity = 97.1%, retention time = 1.28 min.

*(E)-3-(Benzo[d][1,3]-6-dioxol-5-yl)-N-(1-hydroxy-1,3-dihydrobenzo[c][1,2]oxaborol-6-yl)acrylamide (***5g**). Pale yellow solid. Yield = 38%. m.p.: 145–148 °C. ^1^H-NMR (400 MHz, DMSO-*d_6_*) δ_H_ (ppm): 10.64 (1H, s, B-OH), 9.30 (1H, s, NH), 8.47 (2H, s, Ar-H7), 8.24 (1H, d, *J* = 8.0 Hz, Ar-H7), 8.14 (1H, s, Ar-He), 7.81 (1H, d, *J* = 6.3 Hz, Ar-4), 7.76 (1H, d, *J* = 7.6 Hz, Ar-i), 7.71 (1H, d, *J* = 16.0 Hz, Hb), 7.37 (1H, d, *J* = 8.0 Hz, Ar-Hh), 7.20 (1H, d, *J* = 16.0 Hz, Hc), 4.98 (2H, s, H3), 1.98 (2H, s, Hk). ^13^C-NMR (75 MHz, DMSO-*d_6_*) δ_C_ (ppm):170.7, 163.0, 149.4, 148.8, 138.4, 137.8, 134.4, 130.8, 125.9, 124.2, 122.1, 121.5, 70.1, 60.1. ESI-HRMS *m/z* calcd for C_17_H_14_BNO_5_ 323.0965, found 325.0995 [M + H]^+^. HPLC psurity = 100%, retention time = 1.40 min.

### 3.3. In Vitro Antitrypanosomal Assay

*Trypanosoma brucei brucei* 427 trypomastigotes were cultured in Iscove’sss Modified Dulbecco’s medium (IMDM, Lonza, Basel Switzerland) supplemented with 10% fetal calf serum, HMI-9 supplement [56], hypoxanthine, and penicillin/streptomycin at 37 °C in a 5% CO_2_ incubator. Serial dilutions of test compounds were incubated with the parasites in 96-well plates for 24 h and residual parasite viability in the wells determined by adding 20 μL resazurin toxicology reagent (Sigma-Aldrich) and incubating for an additional 2–4 h. Reduction of resazurin to resorufin by viable parasites was assessed by fluorescence readings (excitation 560 nm, emission 590 nm) in a Spectramax M3 plate reader (Molecular Devices, San Jose, CA, USA). Fluorescence readings were converted to % parasite viability relative to the average readings obtained from untreated control wells. IC_50_ values were determined by plotting % viability vs. log[compound] and performing non-linear regression using GraphPad Prism (v. 5.02) software [57].

### 3.4. In Vitro Cytotoxicity Assay

HeLa cells (Cellonex, Johannesburg, South Africa) seeded in 96-well plates were incubated with 20 μM test compounds for 24 h and cell viability assessed using a resazurin fluorescence assay as previously described [57].

### 3.5. In Vitro Antitrichomonal Assay and Mucosal Normal Flora Susceptibility Assay

Axenic cultures of the *Trichomonas vaginalis* G3 strain (the genome strain) were grown in 5 mL TYM Diamond’s medium (containing equine serum and antibiotics) in a 37 °C incubator for 24 h. One hundred millimolar stocks of the compounds were made by dissolving in DMSO and were screened against G3 stain of *T. vaginalis*. Cells that are untreated and treated with 5 μL DMSO (0.1% final concentration) were used as controls. Five milliliters of the 100 mM stocks of the compound library were added for a final concentration of 50 mM. Results were calculated based on manual counts utilizing a hemocytometer after 24 h (parasite motility being the criteria for viability). The IC_50_ values were determined by titration of the stock solutions and hemocytometer counting as described above. Data were plotted on GraphPad Prism (GraphPad Software Inc., San Diego, CA, USA) and theoretical IC_50_ values were determined. To confirm the IC_50_ values, the predicted values were then re-tested as described above. Non-pathogenic mucosal normal flora strains such as *Lactobacillus reuteri* (ATCC 23272, *Lactobacillus acidophilus* (ATCC 43560), and *Lactobacillus rhamnosus* (ATCC 53103) were cultured in Lactobacilli MRS at 37 °C under anaerobic conditions. One hundred milliMolar stock solutions as well as vehicle control DMSO were diluted to 100 μM in media and incubated with empty BDL-sensi-discs (6 mm) for 20 min at room temperature. Discs containing vehicle control, compounds, or various antibiotic discs [levofloxacin (5 μg), gentamicin (10 μg), and gentamicin (120 μg)] were placed onto the bacterial streaked agar plates and incubated overnight at 37 °C. Vehicle, compound, or antibiotic sensitivity was determined via measurement of zones of inhibition around each disc in mm.

### 3.6. In Vitro Antimycobacterial Assay

The minimum inhibitory concentration (MIC) was determined using the standard broth micro dilution method, where a 10 mL culture of *Mycobacterium tuberculosis* pMSp12:GFP [58], was grown to an optical density (OD600) of 0.6–0.7. The media used were: (i) Gaste-Fe (glycerol-alanine-salts) medium pH 6.6, supplemented with 0.05% Tween-80 and 1% Glycerol, and (ii) 7H9 supplemented with 10% Albumin Dextrose Catalase supplement (ADC), 0.05% Tween-80 [59,60]. Cultures grown in Gaste-Fe were diluted 1:100, and cultures grown in 7H9 ADC were diluted 1:500, prior to inoculation of the MIC assay. The compounds to be tested are reconstituted to a concentration of 10 mM in DMSO. Two-fold serial dilutions of the test compound are prepared across a 96-well micro titre plate, after which, 50 μL of the diluted *M. tuberculosis* cultures were added to each well in the serial dilution. The plate layout was a modification of the method previously described [61]. Assay controls used were a minimum growth control (Rifampicin at 2 × MIC), and a maximum growth control (5% DMSO). The micro titre plates were sealed in a secondary container and incubated at 37 °C with 5% CO_2_ and humidification. Relative fluorescence (excitation 485 nM; emission 520 nM) was measured using a plate reader (FLUOstar OPTIMA, BMG LABTECH, Ortenberg, Germany), at day 7 and day 14. The raw fluorescence data were archived and analyzed using the CDD Vault from Collaborative Drug Discovery, in which, data were normalized to the minimum and maximum inhibition controls to generate a dose response curve (% inhibition), using the Levenberg-Marquardt (Burlingame, CA, USA www.collaborativedrug.com) damped least squares method, from which the MIC_90_ was calculated. The lowest concentration of drug that inhibits growth of more than 90% of the bacterial population was considered the MIC_90_.

## 4. Conclusions

In summary, the current drugs used to treat sleeping sickness have serious side effects, and in most cases, showed poor efficacy. Therefore, there is a need to search for new and better treatment options. Benzoxaborole-containing lead compounds have demonstrated promising efficacy and desirable drug-like properties. Due to high attrition in clinical development, it is essential to replete the drug development pipeline with structurally diverse leads. In this study, a small set of cinnamoyl-oxaborole molecules was synthesized and evaluated for trypanocidal activity; and for activity against other prevalent human pathogens. Compound **5g** emerged as new hit with improved activity compared to compound **2** against *T. b. brucei*. Against other pathogens evaluated in this study, these compounds displayed moderate to weak activity without any obvious cytotoxic effect.

## Figures and Tables

**Table 1:** In vitro antimycobacterial, antitrichomonas, and cytotoxicity activities of cinnamic acid-benzoxaborole hybrids.

Compound	IC_50_ (μM)	MIC_99_ (μM)	%Viability
	*T. vaginalis*	H37Rv	HeLa Cells
**5a**	15.3 ± 0.33	61.8	108.5 ± 5.35
**5b**	41.7 ± 0.23	107	87.0 ± 1.59
**5c**	10.2 ± 0.56	26.5	94.3 ± 28.8
**5d**	12.6 ± 0.28	10.7	89.9 ± 5.12
**5e**	11.7 ± 0.28	18.7	102.5 ± 2.34
**5f**	˃50	˃125	109.9 ± 0.84
**5g**	22.8 ± 1.02	40.7	131.5 ± 26.6
**EM**	-	-	3.195
**ME**	0.53	-	-
**RE**	-	0.003	-

EM = emetine, ME = metronidazole, RE = rifampicin.

**Table 2:** In vitro antitrypanosomal activities of cinnamic acid-benzoxaborole hybrids.

Compound	IC_50_ (μM)
**1**	0.292 ± 0.019 ^a^
**2**	0.14 ^b^
**5a**	0.13 ± 0.02
**5b**	0.13 ± 0.01
**5c**	0.92 ± 0.04
**5d**	0.47 ± 0.04
**5e**	0.66 ± 0.03
**5f**	8.71 ± 0.65
**5g**	0.086 ± 0.002
**PE**	0.0012 ± 0.0001

^a^ IC_50_ in μg/mL from reference 33, ^b^ IC_50_ in μg/mL from reference 38, **PE** = pentamidine.

## Supplementary Materials

The following are available online (Synthesis of compound **6**, ^1^H and ^13^C-NMR spectra of **5c**, **5d**, and **5g**).
