# Supplementary material for: Cinnamoyl-Oxaborole Amides: Synthesis and Their in Vitro Biological Activity
Source: Molecules. 2018 Aug 15;23(8):2038. doi: 10.3390/molecules23082038 (PMC6222898; doi:10.3390/molecules23082038)
Supplement: Supplementary file 1 [file molecules-23-02038-s001.pdf]

Cinnamoyl-oxaborole amides: Synthesis and their *in vitro* biological activity

Maureen Gumbo[1], Richard M. Beteck[1], Tawanda Mandizvo[1], Ronnett Seldon[2], Digby F. Warner[3,4], Heinrich C. Hoppe[5,6], Michelle Isaacs[6], Dustin Laming[6], Christina C. Tam[7], Luisa W. Cheng[7], Nicole Liu[8], Kirkwood M. Land[8] and Setshaba D. Khanye[1,6,*]

[1] Faculty of Science, Department of Chemistry, Rhodes University, Grahamstown 6140, South Africa

[2] Drug Discovery and Development Centre (H3-D), Department of Chemistry, University of Cape Town, Rondebosch 7701, South Africa

[3] MRC/NHLS/UCT Molecular Mycobacteriology Research Unit, Department of Pathology, University of Cape Town, Rondebosch 7701, South Africa

[4] Institute of Infectious Disease and Molecular Medicine, University of Cape Town, Rondebosch 7701, South Africa

[5] Faculty of Science, Department of Biochemistry and Microbiology, Rhodes University, Grahamstown 6140, South Africa

[6] Centre for Chemico- and Biomedicinal Research, Rhodes University, Grahamstown 6140, South Africa

[7] Foodborne Toxin Detection and Prevention Research Unit, Agricultural Research Service, United States Department of Agriculture, Albany, CA USA

[8] Department of Biological Sciences, University of the Pacific, Stockton, CA USA

* Correspondence: s.khanye@ru.ac.za (SDK); Tel.: +27-46-603-8717

SUPPLEMENTARY DATA I

1.1 Synthesis of 6-aminobenzo[c][1,2]oxaborol-1(3H)-ol, 7 [1,2]

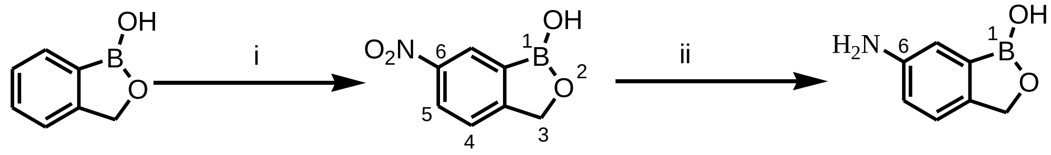

6 **6a** **7**

Reagents and conditions: (i) 99 % HNO_3, -30 ˚C, 3 h; (ii) H_2, Pd/C, rt, 2 h.

(i) ***Nitration***: Benzoxaborole **6** (1.0 g, 4.9 mmol) was added portion-wise to 4.8 mL of fumic nitric acid (99%) maintained at -40 °C and the mixture was stirred between -30 °C and -40 °C for 3 h. Ice and water (20 mL) were added to the mixture and the resultant reaction

mixture stirred at room temperature for an additional 20 minutes. Solid precipitate was filtered and washed with water to give **6a** as a pale yellow solid.

(ii) ***Reduction***: 6-Nitrobenzoxaborole **6a** (0.1 g, 0.56 mmol) was dissolved in ethanol (2 mL) followed by addition of 10% palladium on carbon (0.04 g). The mixture was vacuumed three times to remove air and backfilled with hydrogen. The reaction mixture was allowed to stir at room temperature for 2 h, filtered, and the filtrate was dried under reduced pressure to produce **7** as a brick red solid.

6-Nitrobenzo[c][1,2]oxaborol-1-(3H)-ol, 6a

Yield = 89%. ^{1}H NMR (300 MHz, DMSO-d_6) δ_H (ppm): 9.59 (1H, s, BOH), 8.56 (1H, d, *J* = 1.2 Hz, Ar-H7), 8.31 (1H, dd, *J* = 2.3, 6.2 Hz, Ar-H5), 7.69 (1H, d, *J* = 8.4 Hz, Ar-H4), 5.11 (2H, s, H3).

6-Aminobenzo[c][1,2]oxaborol-1-(3H)-ol, 7

Yield = 91%. ν_{max} = 3244 (NH), 3028 (OH), 1738 (CH). ^{1}H NMR (DMSO-d_6) δ_H (ppm): 9.59 (1H, s, BOH), 8.56 (1H, d, *J* = 1.2 Hz, Ar-H7), 8.31 (1H, dd, *J* = 2.3, 6.2 Hz, Ar-H5), 7.69 (1H, d, *J* = 8.4 Hz, Ar-H4), 4.99 (2H, br, NH), 4.82 (2H, s, H3). ESI-HRMS *m*/*z* calcd for $C_7H_8BNO_2$ 148.9549, found 149.9654 $[M+H]^+$.

References

1. Joardar, S.; Bhattacharyya, A.; Das, S. A palladium on carbon catalyzed one-pot synthesis of substituted benzyimidazoles. Synthesis. 2014, 46, 3121-3132.
2. Joardar, S.; Bhattacharyya, A.; Das, S. A palladium on carbon catalyzed one-pot synthesis of substituted benzyimidazoles. *Synthesis*. **2014**, *46*, 3121-3132.

APPENDIX: NMR spectra of compounds 5c, 5d and 5g

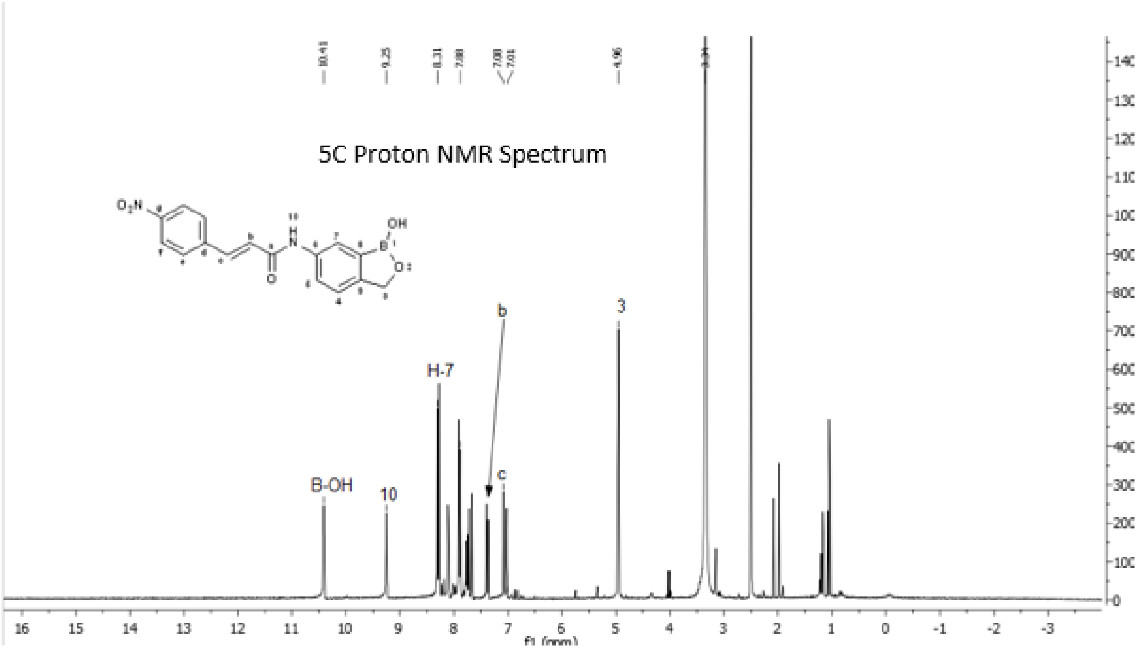

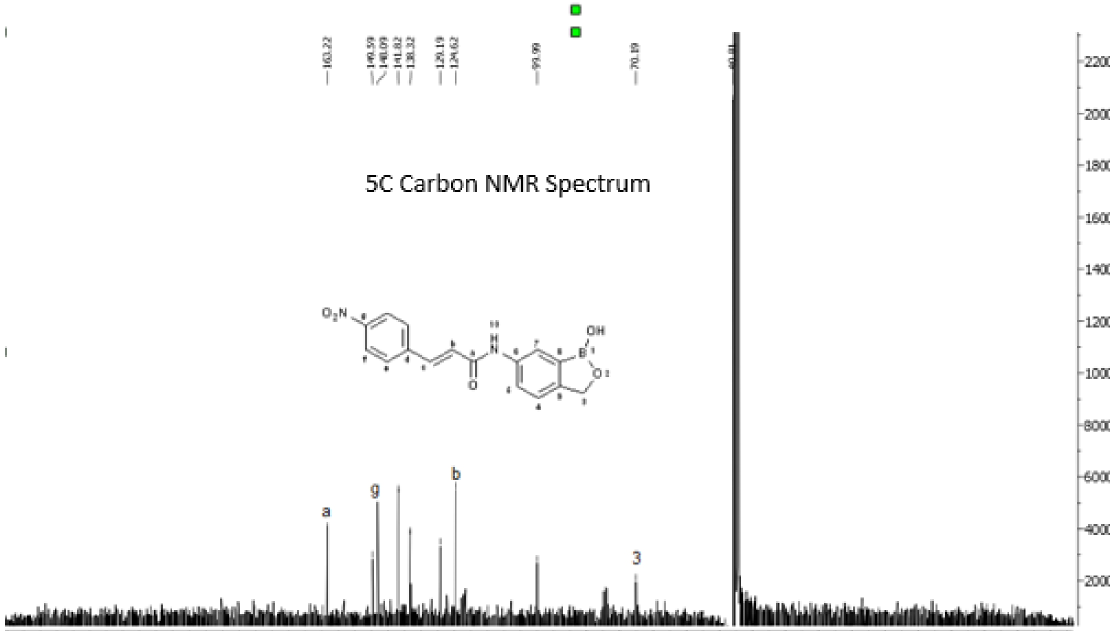

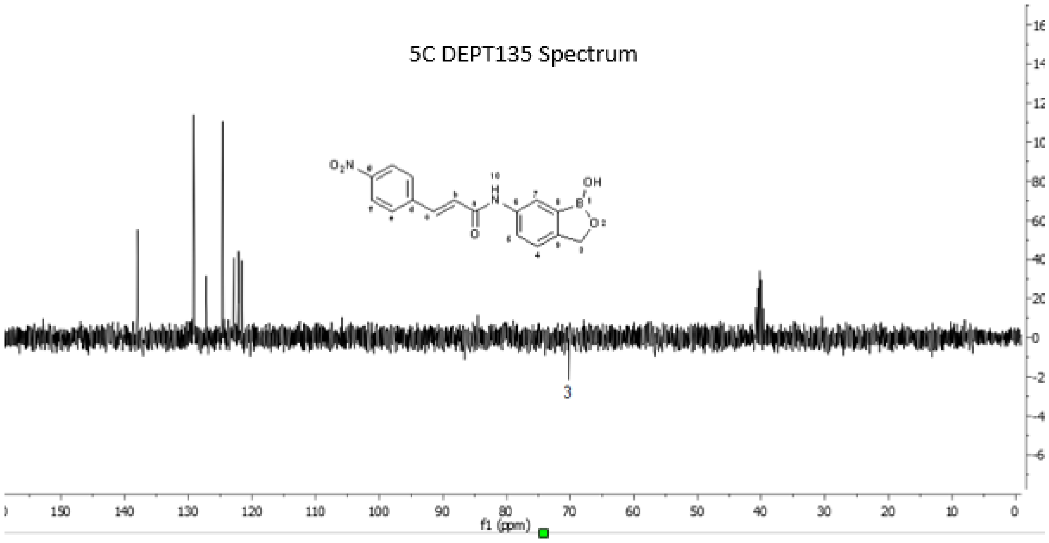
5C DEPT135 Spectrum
3
f1 (ppm)

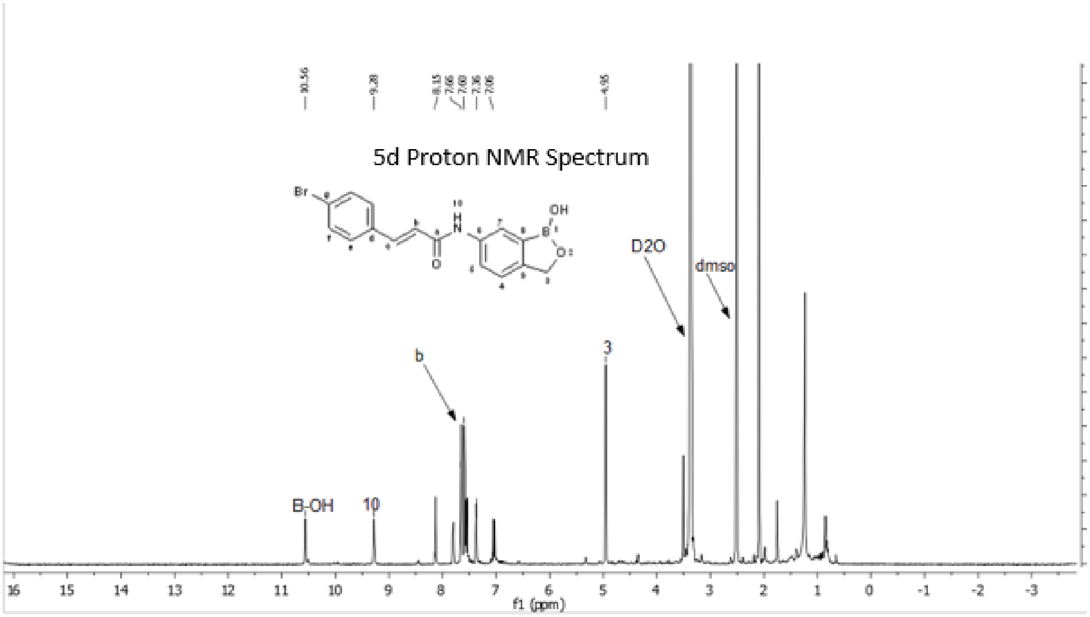
5d Proton NMR Spectrum
B-OH
10
b
3
D2O
dmso
f1 (ppm)

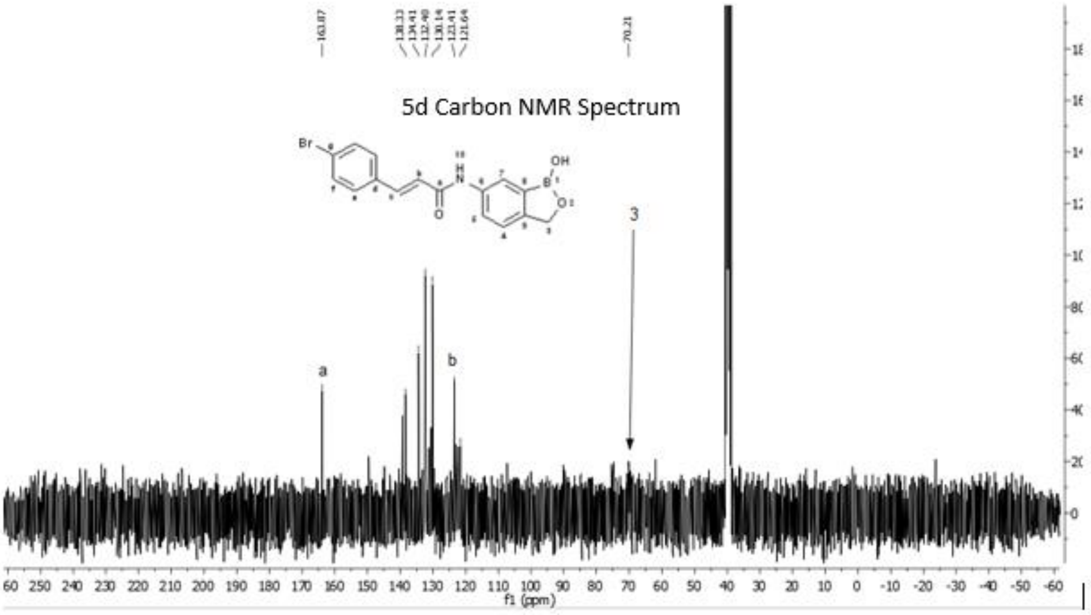
5d Carbon NMR Spectrum
163.87
138.33
134.41
132.40
130.14
123.41
121.64
70.21
a
b
3
f1 (ppm)

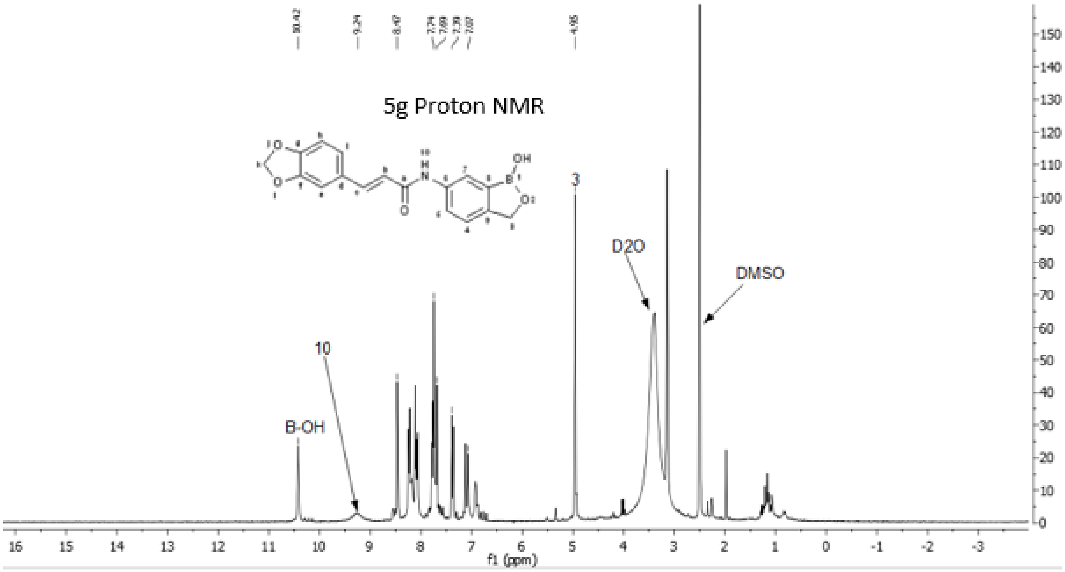
5g Proton NMR
10.42
9.24
8.47
7.74
7.69
7.39
7.07
4.95
B-OH
10
3
D2O
DMSO
f1 (ppm)

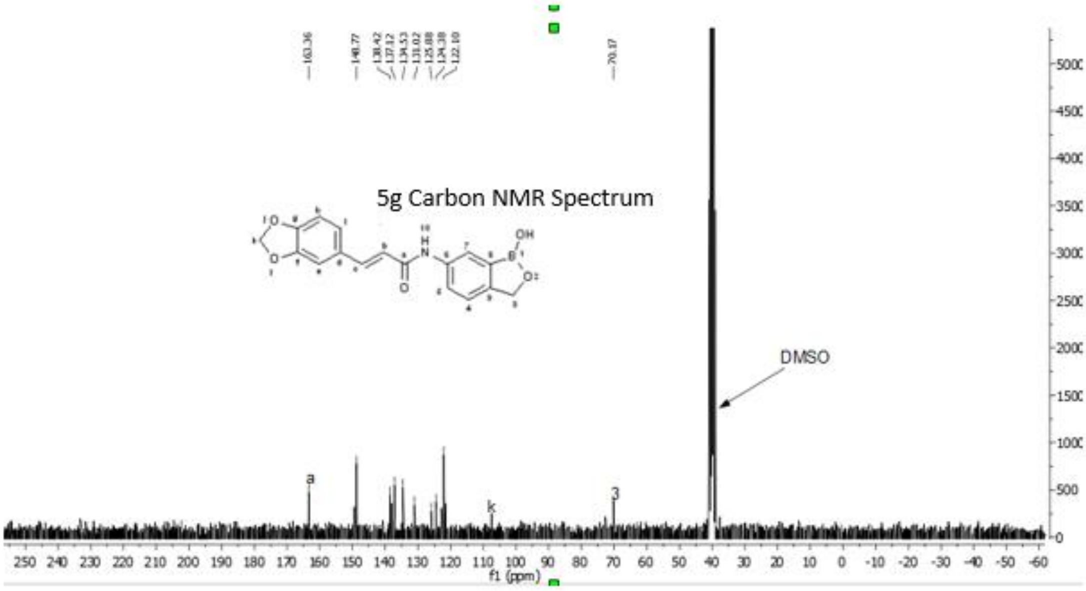

5g Carbon NMR Spectrum
DMSO
f1 (ppm)
